# Protocol for predicting host-microbe interactions and their downstream effect on host cells using MicrobioLink

**DOI:** 10.1016/j.xpro.2024.103570

**Published:** 2025-01-17

**Authors:** Lejla Gul, Anna Julia Elias, Tanvi Tambaku, Marton Olbei, Emily Watters, Balazs Bohar, Dezso Modos, Matthew Madgwick, Tamas Korcsmaros

**Affiliations:** 1 Department of Metabolism, Digestion and Reproduction, Faculty of Medicine, Imperial College London, London W12 0NN, UK; 2 Quadram Institute Bioscience, Norwich, Norfolk NR4 7UQ, UK; 3 Department of Morphology and Physiology, Faculty of Health Sciences, Semmelweis University, 1086 Budapest, Hungary; 4 Earlham Institute, Norwich, Norfolk NR4 7UZ, UK

**Keywords:** Bioinformatics, Cell Biology, Microbiology, Systems biology

## Abstract

Analyzing host-microbe interactions is essential for understanding how microbiota changes disrupt host homeostasis. Here, we present a protocol for predicting host-microbe protein-protein interactions and their downstream effects using MicrobioLink. We describe steps for setting up the environment, installing software, and preparing human transcriptomic and bacterial proteomic data. The protocol outlines procedures for predicting protein-protein interactions through domain-motif interactions, integrating multi-omic datasets to map downstream effects, performing network analyses to identify key regulatory pathways, and visualizing multi-layered networks for systems-level data interpretation.

For complete details on the use and execution of this protocol, please refer to Gul et al.^1^ and Poletti et al.^2^

## Highlights

• Predict host-microbe protein-protein interactions using domain-motif interaction data
• Integrate multi-omic datasets to map downstream effects on host signaling pathways
• Perform network analysis to reveal key regulatory pathways influenced by microbes
• Visualize interaction networks using Cytoscape for systems-level result interpretation

## Abstract (editor highlights)

Publisher’s note: Undertaking any experimental protocol requires adherence to local institutional guidelines for laboratory safety and ethics.

## Graphical abstract

**Figure:**
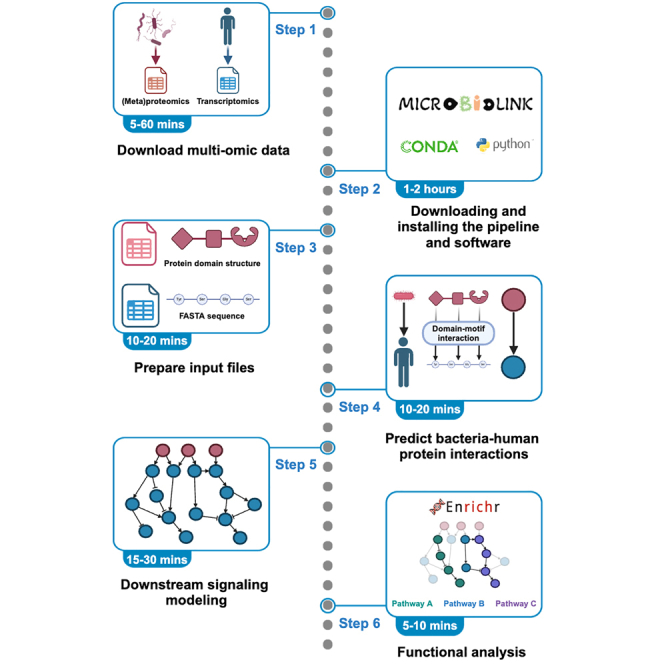

## Before you begin

The protocol below describes a detailed step-by-step guideline to predict host-microbe protein-protein interactions and analyzes their downstream impact on the host cellular signaling network. Setting up the computational environment is crucial to ensure compatibility and smooth execution of the MicrobioLink pipeline. Proper configuration of hardware, software, and required packages minimizes potential errors during the analysis and optimizes performance, particularly when handling large biological datasets. Further details in the ‘system requirement’ subsection. Additionally, the ‘downloading and installing the pipeline and the required software*’* section outlines steps for localizing the pipeline and explain the process for setting up the necessary environment. It also details how to download and install essential software tools.

***Note:*** To effectively visualize networks generated by this analysis, ensure that Cytoscape version 3.10.2 is installed on your system. Cytoscape will be used to create interactive graphical representations of host-microbe interaction networks, facilitating interpretation of the results.

***Note:*** For general troubleshooting issues related to software or package unavailability, missing parameters, empty output files, or directory errors, please refer to the “troubleshooting 1, 2, 3, 4, 5, 6, and 7” section at the end of this protocol. This section provides solutions to common setup and execution problems.

### System requirement

MicrobioLink is compatible with Unix/Linux/Windows operating systems. To ensure optimal performance, we recommend certain hardware specifications. A minimum of 8 GB of RAM is recommended, with 16 GB preferred for large-scale analyses, as adequate memory helps prevent slowdowns or crashes during network analyses. At least 1 GB of free disk space is needed to store input, intermediate, and output files, through larger dataset may require additional storage. Finally, for network visualization in Cytoscape or other visualization tools, a system equipped with modern integrated graphics ensures smooth rendering of large or complex networks.

The MicrobioLink pipeline depends on a range of Python libraries and bioinformatics tools for handling large datasets, performing statistical computations, and visualizing complex networks. Installing the correct versions of these packages will help avoid compatibility issues and ensure that the pipeline runs efficiently. For data manipulation and numerical operations **pandas** (v.2.2.2), **numpy** (v.1.26.4), and **scipy** (v.1.13.1) are essential. **MyGene (**v.3.2.2) provides fast access to gene annotations, while **omnipath** (v.1.0.8) offers the primary knowledge network of protein-protein interactions, essential for the downstream network analysis. **Pyfasta** (v.0.5.2) and **biopython** (v.1.84) facilitate the handling and processing of FASTA files, which are standard in bioinformatics for storing protein and nucleotide sequences. Finally, **Cytoscape** (v.3.10.1) is required for visualizing the interaction networks generated by the pipeline, allowing users to interpret host-microbe interaction patterns and downstream effects visually.

### Downloading multi-omic data for the case study

**Timing: 5–60 min (depending on the size of the datasets)**

MicrobioLink combines host transcriptomics and bacterial proteomics that enables a more comprehensive analysis of host-microbe interactions, linking microbial influence directly to changes in gene expression and protein function in the host. For the case study, we use public single-cell data from^3^ and proteomic data derived from *Bacteroides thetaiotaomicron* extracellular vesicles published by Gul and colleagues.^1^ All necessary input data for running this protocol are available in the GitHub repository. (https://github.com/korcsmarosgroup/MicrobioLink2).

***Note:*** Human transcriptomic data provides gene expression profiles, which are crucial for identifying how microbial interactions might influence gene regulation within the host. The pipeline uses average gene expression counts from processed single-cell or bulk RNA-seq data, enabling downstream analysis to focus on potential gene expression changes.

***Note:*** The endpoint file provides a focus for the analysis by defining the target genes that the pipeline seeks to connect to bacterial proteins, with the aim of exploring which signaling pathways may be perturbed to influence these specific genes. The endpoint file can contain genes that are differentially expressed between conditions (p-value < 0.05) along with their fold change values. In this case, MicrobioLink identifies signaling pathways potentially impacted by bacterial interactions that could influence differential gene expression. Alternatively, the endpoint file may include a list of genes derived from a single condition. For example, it could contain genes encoding secreted ligands described in inflamed condition. In this case, MicrobioLink assesses how bacterial interactions might influence cellular communication mediated through these secreted ligands.

***Note:*** Bacterial proteomics includes proteins from the bacterial proteome, representing the microbial components that may interact with host proteins. The pipeline accepts either a list of bacterial UniProt IDs or a UniProt Proteome (UP) ID, which allows the pipeline to download and analyze the complete proteome of a bacterial strain. The UP option is particularly useful for large-scale analyses where all potential proteins from a specific bacterial strain are of interest. Analyzing these interactions across bacterial strains provides insights into the broader effects of microbes on host cellular processes, including both pathogenic and probiotic effects.

### Downloading and installing the pipeline and the required software

**Timing: 1–2 h**

Main steps for downloading and installing the pipeline and required software.

1. Download Anaconda (recommended).

***Note:*** Anaconda is a widely used package and environment manager that simplifies the installation and management of required software packages. Details on installation are described here: https://docs.anaconda.com/free/anaconda/install/index.html.

***Alternatives:*** If users cannot use the Conda environment, proceed directly with Python environment setup as described below.

2. Download the MicrobioLink pipeline from GitHub (https://github.com/korcsmarosgroup/MicrobioLink2).
  a. Download the zip archive and extract it, or use the git-clone command:

>git clonehttps://github.com/korcsmarosgroup/MicrobioLink2.git

***Note:*** Ensure that the microbiolink_env.yml file, which specifies required packages, is located within the workflow folder of the repository. This file will facilitate the automatic installation of compatible software versions within the environment.

3. Set up the environment
  a. Using Anaconda (recommended).
    i. To create a Conda virtual environment with the required packages, navigate to the downloaded MicrobioLink2 directory and execute the following commands:

>cd MicrobioLink2

>conda create --name microbiolink --file workflow/microbiolink_env.yml

***Note:*** Before the environment initiation, it is essential to have Conda installed on the system, details are described in Step 1.

***Optional:*** For users unable to use Conda, create a Python environment using ‘venv‘ and install each package individually using pip, packages are listed in the ‘*workflow/microbiolink_env.yml*’:

>python3 -m venv microbiolink_env

4. Activate the environment:
  a. Using Anaconda (recommended).
    i. Open the Anaconda terminal by going to the "Environments" – select the appropriate environment and click "Open Terminal".

>conda activate microbiolink

***Optional:*** For users unable to use Conda, use the following command in Terminal/Command Line to activate the virtual environment:

>source microbiolink_env/bin/activate # On Windows: `microbiolink_env\Scripts\activate`

## Key resources table

**Table:** 

REAGENT or RESOURCE	SOURCE	IDENTIFIER
**Deposited data**		

Human single-cell RNA-seq data (processed)	Kong et al.^3^	https://github.com/korcsmarosgroup/MicrobioLink2/tree/main/case_study_input/input/human_protein
Bacterial proteomics	Gul et al.^1^	https://github.com/korcsmarosgroup/MicrobioLink2/tree/main/case_study_input/input/bacterial_protein

**Software and algorithms**		

Pandas v.2.2.2	PyData	https://pandas.pydata.org; RRID:SCR_018214
Numpy v.1.26.4	Harris et al.^4^	http://www.numpy.org; RRID:SCR_008633
Scipy v.1.13.1	Virtanen et al.^5^	http://www.scipy.org/; RRID:SCR_008058
Mygene v.3.2.2	Wu et al.^6^	RRID:SCR_018660
OmniPath v.1.0.8	Turei et al.^7^	https://omnipathdb.org/
Gget v.0.28.6	Luebbert et al.^8^	https://github.com/pachterlab/gget
Pyfasta v.0.5.2	https://pypi.org/project/pyfasta/0.2.9/	https://github.com/brentp/pyfasta
Biopython v.1.84	Cock et al.^9^	http://biopython.org; RRID:SCR_007173
Python v.3.9	Python	https://www.python.org/; RRID:SCR_008394
Cytoscape v.3.10.1	Shannon et al.^10^	https://cytoscape.org; RRID:SCR_003032

**Other**		

Code to reproduce the analysis	This publication	https://github.com/korcsmarosgroup/MicrobioLink2/tree/main/workflow
Computer with Mac/Linux/Windows operating system and internet access	N/A	N/A
At least 8 GB RAM (16 GB preferred), ≥1 GB free disk space	N/A	N/A
Anaconda environment for package management (optional)	https://docs.anaconda.com/free/anaconda/install/index.html	https://www.anaconda.com/; RRID:SCR_025572

## Step-by-step method details

All parts of the MicrobioLink pipeline can be run from the terminal with the necessary command-line argument defined by each script. Each parameter is defined with a specific help message, providing users with guidance on the expected format and purpose of the argument. Example usage of each step is shown using files from the provided GitHub repository (“*case_study_input/input*” and “*case_study_output/output*” folders), allowing users to follow along with a real use case. The following sections include step-by-step instructions and visual examples to ensure users can accurately format their inputs and avoid common errors.

### Preparing input files for the MicrobioLink pipeline

**Timing: 10–20 min(depending on the size of data)**

To ensure accurate predictions of host-microbe interactions and downstream effects, input files should be carefully prepared and formatted to meet the requirements of the pipeline. Below, we outline the preparation steps for each input file.

1. Z-score filtering for human transcriptomic data (z-score_filter_terminal.py).
  ***Note:*** This step involves filtering gene expression data to reduce noise by excluding lowly expressed genes. Filtering gene expression data to exclude genes below a set Z-score threshold ensures that only genes with relatively high expression levels are included in the analysis. This script takes bulk or single-cell gene expression data in normalized average expression count matrix format as input.
  ***Note:*** The script performs log2 transformation, filters out lowly expressed genes based on z-score cut-off, and outputs the filtered results into a CSV file.
  ***Optional:*** This step may be skipped if the user does not want to filter lowly expressed genes, or if the necessary filtering has already been performed.
  a. Run the script with the necessary command-line arguments.
    >cd MicrobioLink2/workflow
    >python z-score_filter_terminal.py --input_file “case_study_input/input/human_transcriptomics/colon_BEST4_enterocyte_CD.csv” --output_file “case_study_input/input/human_transcriptomics/enterocyte_colon_CD_zscore.csv” --zscore -3
    ***Note:*** The parameter **"--input_file**" is required and is used to specify the path for the input CSV file that contains the gene expression data in the first column (gene symbol or UniProt) and normalized average expression counts in subsequent columns (Figure 1).
    ***Note:*** The parameter "**--output_file**" is required to specify the name and path for the output CSV file where filtered results will be saved.
    ***Note:*** The "**--zscore**" parameter is used to set a Z-score cut-off for filtering out lowly expressed genes in the dataset. If this parameter is not provided, it defaults to −3.^11^ By adjusting this parameter, users can control the threshold for filtering based on gene expression levels. The parameter expects a numeric value (e.g., −3, −2.5, −1), typically representing the number of standard deviations below the mean expression level, with higher values filtering out more genes.
    ***Note:*** Z-score filtering only works well for data with a normal distribution. If the data does not follow normal distribution, the least 10% of expressed genes may be excluded from the analysis. It is the user’s responsibility to assess this.

**Figure 1:**
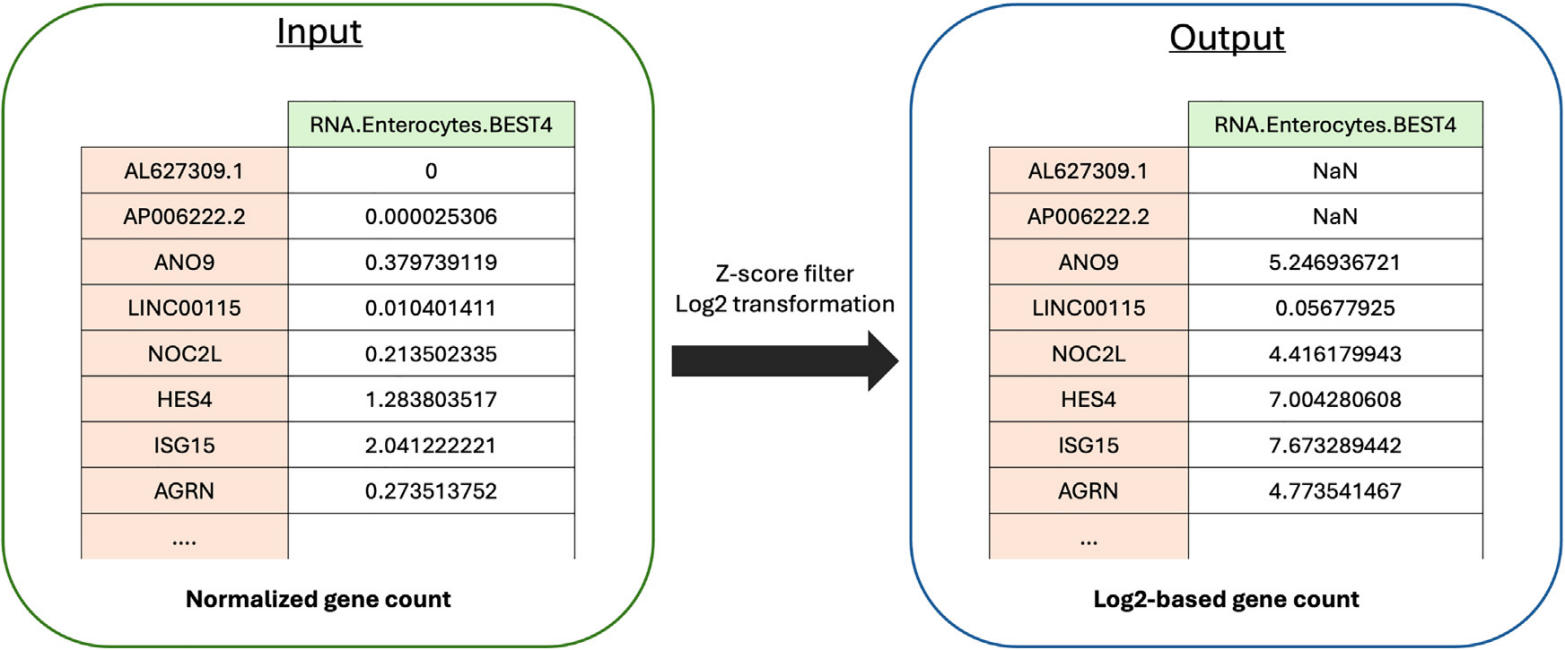
Transformation of normalized gene counts to Log2-based values with Z-score filtering
2. Obtain the human protein FASTA file (get_human_fasta.py).
  ***Note:*** This step retrieves protein sequences for human genes in FASTA format. This file is essential for the protein interaction predictions, as it provides the sequences used to identify domain binding motifs and predict host-microbe interactions.
  ***Note:*** The script offers a filtering for membrane-based or secreted proteins that is crucial when analyzing host-microbe interactions involving extracellular bacteria, as their interactions with the host typically occur through the cell membrane.
  a. Run the script with the necessary command-line arguments.
    >cd MicrobioLink2/workflow
    >python get_human_fasta.py --gene_expression “case_study_input/input/human_transcriptomics/enterocyte_colon_CD_zscore.csv” --id_type "genesymbol" --sep "," --location_filter_list [plasma_membrane_transmembrane plasma_membrane_peripheral] --output_folder “case_study_input/input/human_transcriptomics/” --output_sequences “protein_sequences.fasta”
    ***Note:*** The "**--gene_expression**" parameter is a required argument that specifies the file path to the transcriptomics data. The Uniprot or gene symbol IDs must be found in the first column of the file while corresponding expression values must be placed in the second column (Figure 2). If the z-score filter is applied then this file is the output of Step 1, otherwise the normalized average gene count matrix (described in the “before you begin” section).
    ***Note:*** The "**--id_type**" parameter is a required argument that specifies the type of gene identifier used in the transcriptomics data file. This parameter restricts input to specific options, offering only two valid choices: "genesymbol" or "uniprot". By setting choices = ["genesymbol", "uniprot"], the program ensures that users select one of these options, avoiding potential errors in data interpretation.
    ***Note:*** The "**--sep**" parameter is a required argument that specifies the field separator (e.g., ";", "\t", or "|") used in the input file, allowing the program to correctly parse and interpret the data.
    ***Note:*** The "**--location_filter_list**" parameter allows users to narrow down protein analysis based on specific cellular locations. This parameter accepts multiple location filters as a space-separated list. If no filter list is provided, the program will default to *None*, meaning no location-based filtering will occur, and all proteins will be included in the analysis.
    ***Note:*** Options for subcellular location filtering are the following: *plasma_membrane_transmembrane* (proteins located within the plasma membrane and spanning across it) and/or *plasma_membrane_peripheral* (proteins associated with the peripheral site of the plasma membrane) and/or *secreted* (proteins that are secreted into the extracellular space). Required format is separated by space and without quotation marks and brackets. E.g.: [plasma_membrane_transmembrane plasma_membrane_peripheral]. Default: None.
    ***Note:*** The "**--output_folder**" parameter is a required argument that designates the folder where the script will save its output files. The default value is ".", which represents the current directory, hence if a specific folder path is not provided, the script will save the output files to the directory from which it was run.
    ***Note:*** The "**--output_sequences"** parameter is a required argument that specifies the filename for saving protein sequences. If no filename is provided, the program will save protein sequences to a file named "*protein_sequences.fasta*" in the specified output folder or the current directory.
    ***Note:*** If the script fails to fetch data for one or more UniProt IDs it prints the message "Failed to fetch data for uniprots: {uniprots}".
    ***Note:*** The following error message may appear if the mygene package is not installed properly: ‘AttributeError: partially initialized module 'charset_normalizer' has no attribute 'md__mypyc'’. Further details and solutions for this issue are provided in the “troubleshooting 6” section.

**Figure 2:**
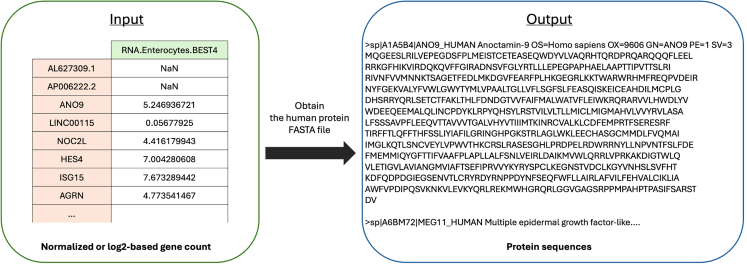
Downloading FASTA sequences for expressed genes
3. Downloading bacterial proteins with their domain structure (download_bacterial_proteins.py).
  ***Note:*** This script downloads bacterial protein domain structures from the UniProt database. These bacterial proteins serve as the microbial component in host-microbe interaction predictions.
  a. Run the script with the necessary command-line arguments.
    >cd MicrobioLink2/workflow
    >python download_bacterial_proteins.py --id_list “case_study_input/input/bacterial_protein/OMV_proteins.csv” --sep "," --id_type "Uniprot" --id_column 1 --output “case_study_input/input/bacterial_protein/BT_BEV_domains.tsv”
    ***Note:*** The "**--id_list**" parameter is a required argument that specifies the file path to an existing file containing a list of identifiers either in UniProt or UniProt Proteome (UP) ID format (Figure 3). Ensure no headers or additional information is included beyond these columns, as alteration may cause compatibility issues. Using the UP option allows for a more comprehensive analysis of potential microbial interactions across all proteins of a strain, especially useful in large-scale studies.
    ***Note:*** The "**--sep**" parameter is a required argument that specifies the field separator used in the input file, helping the script correctly parse and interpret the data. The argument expects a single character (e.g., ";", "\t", or "|") representing the separator between fields in the input file.
    ***Note:*** The "**--id_type**" parameter is a required argument that defines the type of identifier used in the input data - "Uniprot" or "UP".
    ***Note:*** The "**--id_column**" parameter is a required argument that specifies the column number containing the proteome or protein ID within the input file. The argument expects an integer representing the column number in the input file where the proteome or protein IDs are located. Python starts to count at 0, therefore the user-provided column number is automatically decreased by one. User provided "--id_column" 1 would refer to the first column.
    ***Note:*** The "**--output**" parameter is a required argument that specifies the file path and name where the program will save its output.

**Figure 3:**
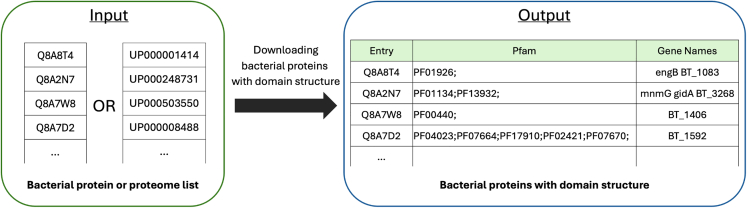
Retrieving domain structures for bacterial proteins

### Predicting interactions between human and microbial proteins based on domain-motif interactions

**Timing: 10–20 min(depending on the size of data)**

This step predicts protein-protein interactions between human and bacterial proteins through domain-motif interactions (DMIs), fundamental points of protein binding and function that enable precise alignment of complementary structures and facilitate biological processes, allowing proteins to communicate, modify each other’s activity, or drive signaling events within cells. By focusing on DMIs involving motifs on disordered protein regions, this approach reduces false positives and ensures that only flexible interaction surfaces, necessary for binding, are kept.

4. Host-microbe protein-protein interaction prediction (DMI.py).
  ***Note:*** This script uses structural data, specifically DMIs, to predict interactions between human and microbial proteins. The analysis is based on *in vitro* verified DMIs from the Eukaryotic Linear Motif (ELM) database, which provides validated motif-domain relationships in eukaryotic proteins (Figure 4).
  ***Note:*** The script utilizes external libraries and modules, such as pyfasta and re, to process and analyze biological sequence data and interaction predictions between human and microbial proteins.
  a. Run the script with the necessary command-line arguments.
    >cd MicrobioLink2/workflow
    >python DMI.py --fasta_file “case_study_input/input/human_transcriptomics/protein_sequences.fasta” --elm_regex_file “case_study_input/input/elm /elm_classes_2020.tsv” --motif_domain_file “case_study_input/input/elm /elm_interaction_domains.tsv” --bacterial_domain_file “case_study_input/input/bacterial_protein/BT_BEV_domains.tsv” --output_file “case_study_output/output/HMI/BT_enterocyte_cd_prediction_output_usecase.csv”
    ***Note:*** The "**--fasta_file**" parameter is a required argument that specifies the path to a FASTA file containing human protein sequences, downloaded in Step 2.
    ***Note:*** The "**--elm_regex_file**" parameter is a required argument that specifies the path to a file containing motif regular expressions from the ELM database. The input file should be in .tsv format. ELM Identifiers are required to be in the second column, while Regex information is in the fifth column. The required table is provided in the GitHub repository or can be downloaded directly from the ELM database: http://elm.eu.org/elms/elms_index.tsv.
    ***Note:*** The "**--motif_domain_file**" parameter is a required argument that specifies the path to a file containing motif-domain interaction data from the ELM database. The input file should be in .tsv format. ELM Identifiers are required to be in the first column, while associated Pfams should be in the second column. The required table is provided in the GitHub repository or can be downloaded directly from the ELM database: http://elm.eu.org/interactions/as_tsv.
    ***Note:*** The "**--bacterial_domain_file**" parameter is a required argument that specifies the path to a file containing bacterial protein domain information. The input file contains Pfams in the second column separated by semicolon. The required file is automatically generated by the "get_bacterial_domain_structure.py" script, details are described in Step 3.
    ***Note:*** The "**--output_file**" parameter is a required argument that specifies the filename and path where the script will save its output file. The header of the output file will be automatically generated as "# Human Protein; Motif; Start; End; Bacterial domain; Bacteria Protein".
    ***Note:*** If the user is not using Python 3.9, the following error message may appear: ‘*Error: cannot import name 'Mapping' from 'collections'*’. Further details and solutions for this issue are provided in the “troubleshooting 5” section.

**Figure 4:**
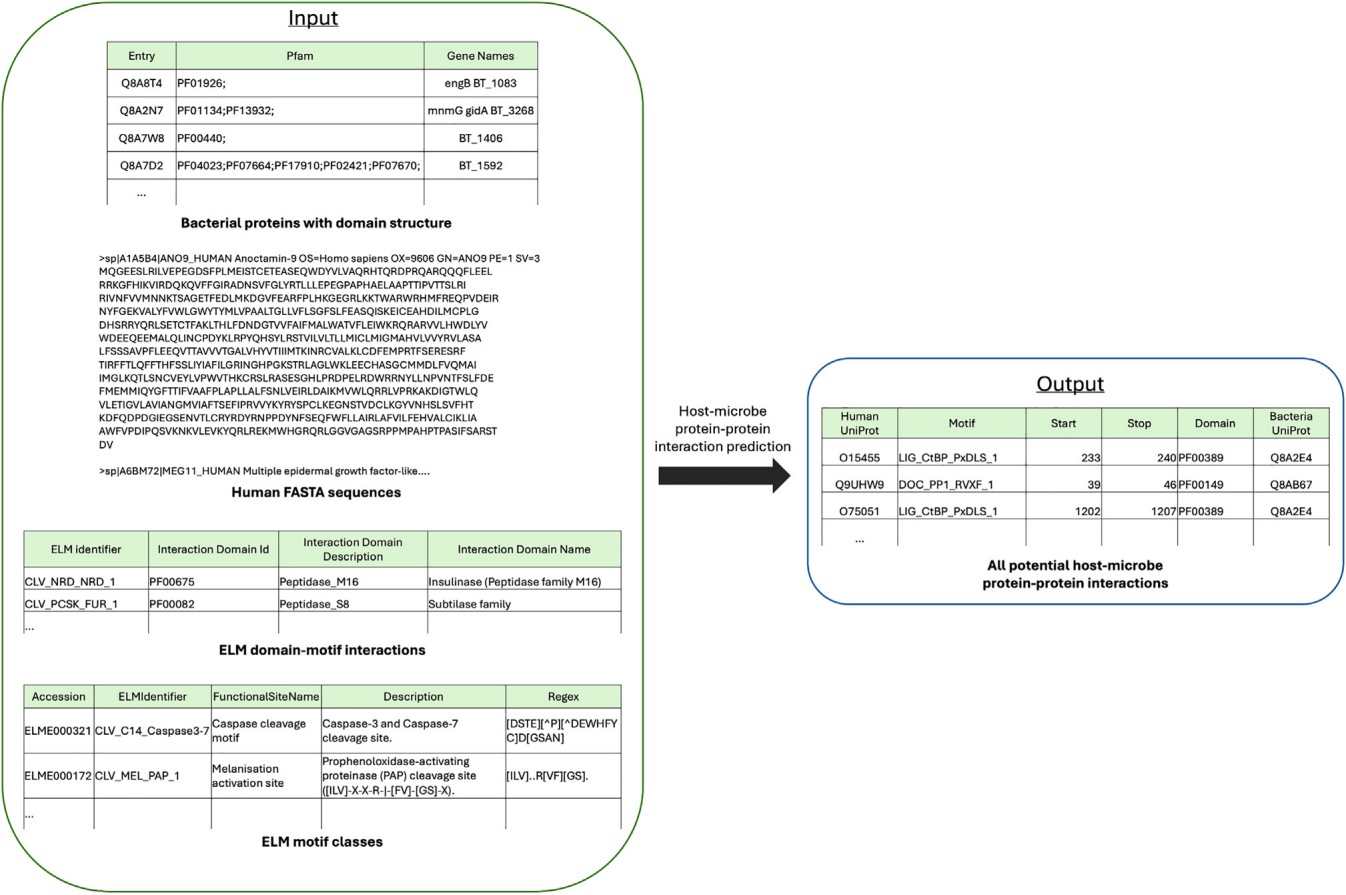
Integrating bacterial protein domains with human protein motifs to predict host-microbe protein-protein interactions
5. Quality control of interactions based on target motif location (idr_prediction.py).
  ***Note:*** This step applies a quality control filter, using the IUPred pipeline,^12^ to exclude interactions where the motifs are located outside disordered regions or within globular domains of the host protein, as these interactions are less likely to be biologically significant^12^ (Figure 5).
  a. Run the script with the necessary command-line arguments.
    >cd MicrobioLink2/workflow
    >python idr_prediction.py --hmi_prediction “case_study_output/output/HMI/BT_enterocyte_cd_prediction_output_usecase.csv” --fasta_file “case_study_input/input/human_transcriptomics/protein_sequences.fasta” --resources “case_study_input/input/” --results “case_study_output/output/” --output “case_study_output/output/HMI/IUPred/BT_enterocyte_idr_cd_usecase.csv”
    ***Note:*** The "**--hmi_prediction**" parameter is a required argument that specifies the path to an existing file containing host-microbe interaction prediction data. The file delimiter must be “;”, human UniProt IDs must be in the first column, while motif name, start, and stop information must be in the second, third, and fourth columns. This structure is automatically generated by the DMI.py script, described in Step 4.
    ***Note:*** The "**--fasta_file**" parameter is a required argument that specifies the path to a FASTA file containing human protein sequences (Step 2).
    ***Note:*** The "**--resources**" parameter is a required argument that specifies the path to a folder containing resources needed by the program (see in Notes). This directory should be a valid, accessible path where the user has ‘*write*’ permission.
    ***Note:*** The "**--results**" parameter is a required argument that specifies the path to the folder where the program will save its output results. This directory should be a valid, accessible path where the user has ‘*write*’ permission.
    ***Note:*** The "**--output**" parameter is a required argument that specifies the file name and path where the program will save its outputs. The header of the output file will be automatically generated as "# Human Protein; Motif; Start; End; Bacterial domain; Bacterial protein".
    **CRITICAL:** The script imports an external script named iupred2a.py. It is included in the GitHub repository (‘*workflow/IUPred’* folder), and it should be placed in the same folder as the idr_prediction.py script.
    **CRITICAL:** The script needs special data required for the disordered region prediction, such as energy matrices and histograms. These files, located in the Github repository (‘*case_study_input/input/iupred_data*’ folder), contain data essential for IUPred prediction of disordered regions in protein sequences.
    ***Note:*** The ‘*anchor2_energy_matrix’* estimates energy changes in protein interfaces, assisting in identifying protein regions that are likely disordered using the Anchor2 tool. The ‘*anchor2_interface_comp’* complements the energy matrix by containing interface comparison data needed by Anchor2.
    ***Note:*** The ‘*iupred2_long_energy_matrix’ and ‘iupred2_short_energy_matrix’* predict disordered regions in proteins, tailored for different sequence lengths (long and short).
    ***Note:*** The ‘*long_histogram’ and ‘short_histogram’* provide statistical distributions that IUPred uses to analyze long and short protein sequences.
    ***Note:*** When running the IUPred function, the script uses "short" as a predefined method type for IUPred, and ANCHOR modeling is set to True. Further details are described in Meszaros et al.^12^

**Figure 5:**
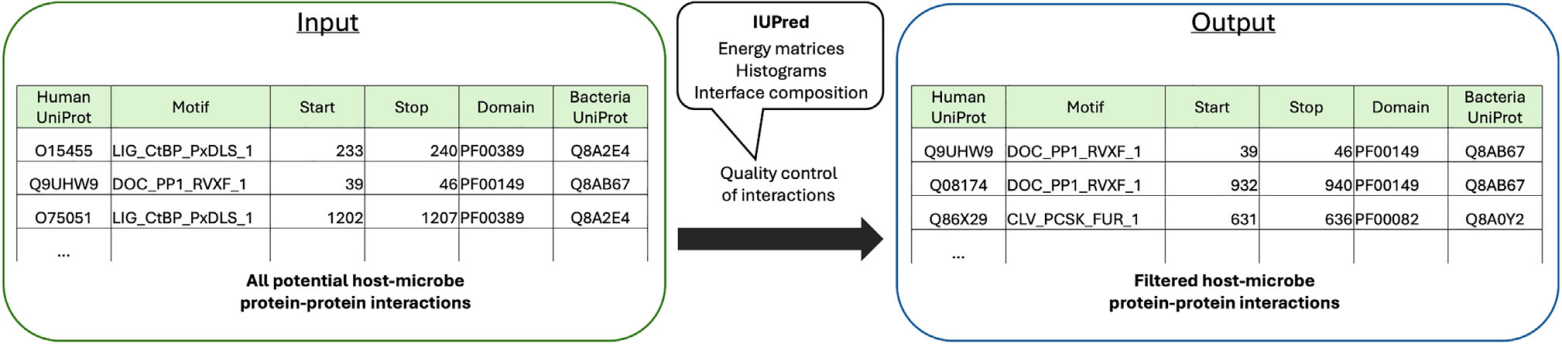
Applying IUPred disordered region filter to refine host-microbe protein-protein interactions

### Downstream signaling modeling

**Timing: 15–30 min(depending on the size of the data)**

This step models the downstream signaling pathways influenced by host-microbe protein-protein interactions, using the interactions identified in Step 5 as perturbation points. By tracing how microbial factors alter entire host cellular networks, including gene regulation, immune responses, and cellular communication, this analysis reveals the cascading effects underlying complex host responses and disease mechanisms. Using the TieDIE (Tied Diffusion Through Interacting Events) tool,^13^ it identifies specific paths connecting bacterial targets to shifts in gene expression and cellular functions.

6. Preparing input files for the TieDIE analysis (tiedie_input_processing.py).
  ***Note:*** TieDIE requires specific input files to map the potential downstream effects of microbial interactions on host cellular signaling pathways. This step involves preparing these files in the correct formats to ensure that the analysis accurately models the downstream cascade initiated by host-microbe interactions (Figure 6).
  a. Run the script with the necessary command-line arguments.
    >cd MicrobioLink2/workflow
    >python tiedie_input_processing.py --transcriptomics_file “case_study_input/input/human_transcriptomics/enterocyte_colon_CD_zscore.csv” --value_column 2 --sep_transcriptomics ";" --endpoint_file “case_study_input/input/human_transcriptomics/Enterocytes BEST4_degs_fc05.csv” --sep_endpoint "," --endpoint_pvalue_column 2--endpoint_pvalue_column 3 --hmi_prediction_file “case_study_output/output/HMI/IUPred/BT_enterocyte_idr_cd_usecase.csv” --output_dir “case_study_output/output/” --upstream_input_filename “usecase_upstream.input” --downstream_input_filename “usecase_downstream.input” --pathway_input_filename “usecase_pathway.sif”
    ***Note:*** The "**--transcriptomics_file**" parameter is a required argument that specifies the path to the file containing transcriptomics data to contextualise the primary knowledge network for the explored tissue (bulk transcriptomics) or cell (single-cell transcriptomics). The Uniprot or gene symbol IDs must be found in the first column of the file while corresponding expression values must be placed in the second column (Figure 6). If the z-score filter is applied then this file is the output of Step 1, otherwise the normalized average gene count matrix (described in the “before you begin” section).
    ***Note:*** The "**--value_column**" parameter is a required argument that specifies the column number in the transcriptomics file where expression values are located. User provided *"--value_column 2*” would refer to the second column.
    ***Note:*** The "-**-sep_transcriptomics**" parameter is a required argument that specifies the separator (e.g., ";", "\t", or "|") used in the transcriptomics data file, enabling the program to correctly parse the data.
    ***Note:*** The "**--endpoint_file**" parameter is a required argument specifying the file path to an endpoint file for the analysis. This file defines where the signaling cascade terminates, representing the final targets affected by host-microbe interactions. It should contain data on differentially expressed genes significantly altered between conditions or a list of genes relevant to specific processes, such as genes involved in immune response or inflammation.
    ***Note:*** The "**--endpoint_pvalue_column**" parameter is an optional argument that specifies the column number in the endpoint file containing the adjusted p-value or FDR value for differentially expressed genes. This column is typically used to filter genes based on statistical significance, retaining only those with p-values below a certain threshold (e.g., 0.05). If not provided (e.g. the endpoint file is not a DEG table but a list of genes of interest), the script will include all genes in the endpoint file. User provided *"-- endpoint_pvalue_column 2*” would refer to the second column.
    ***Note:*** The "**--endpoint_value_column**" parameter is a required argument that specifies the column number in the endpoint file containing the fold change or expression values for (differentially) expressed genes. This data is used in downstream analyses to calculate transcription factor activity. User provided *"-- endpoint_value_column 3*” would refer to the third column.
    ***Note:*** The "**--sep_endpoint**" parameter is a required argument that specifies the separator (e.g., ";", "\t", or "|") used in the endpoint data file, enabling the program to correctly parse the data.
    ***Note:*** The "**--hmi_prediction_file**" parameter is a required argument that specifies the path to an existing file containing filtered host-microbe interaction predictions (Step 5). It serves as the starting point for identifying pathways influenced by microbial interactions. In the input file, the first column should be "#Human Protein”, and the sixth column should be "Bacterial Protein". This format is automatically achieved by using the output of idr_prediction.py script, described in Step 5.
    ***Note:*** The "**--output_dir**" parameter is a required argument that specifies the directory where the script will save all its output files. This directory should be a valid, accessible path where the user has ‘*write*’ permission.
    ***Note:*** The "**--upstream_input_filename**" parameter is a required argument that allows the user to specify a custom name for the upstream input file generated by the program. If the user does not specify this argument, the program will save the upstream input file using this default name ("*upstream.input*") in the specified output directory.
    ***Note:*** The "**--downstream_input_filename**" parameter is a required argument that allows the user to specify a custom name for the downstream input file generated by the script. If the user does not specify this argument, the program will save the downstream input file using this default name ("*downstream.input*") in the specified output directory.
    ***Note:*** The "**--pathway_input_filename**" parameter is a required argument that allows the user to specify a custom name for the pathway input file generated by the program. If the user does not specify this argument, the program will save the pathway input file using this default name (“*pathway.sif*”) in the specified output directory.
    ***Note:*** The outputs of this step include the ‘contextualised_regulator-target_network.txt’ describing a contextualized transcription factor – target gene interaction table derived from CollecTRI highlighting those regulatory interactions where the transcription factor is in the transcriptomics data and the target gene is in the endpoint gene list (details above); the ‘upstream.input’ 3-column tab-separated file that describes the gene name, the input heat, and the expected functional effect of the perturbation (+/−)); the ‘pathway.sif’ 3-column tab-separated file including the <source>, <interaction>, and <target> information; and the ‘downstream.input’ file having a similar structure to upstream.input, but the third column indicates the inferred activity of the gene (rather than the expected effect).
    ***Note:*** If the output file is empty after running the script, it may occur if the input files do not have unified identifiers, such as UniProt IDs, across all files; details are described in the “troubleshooting 7” section.

**Figure 6:**
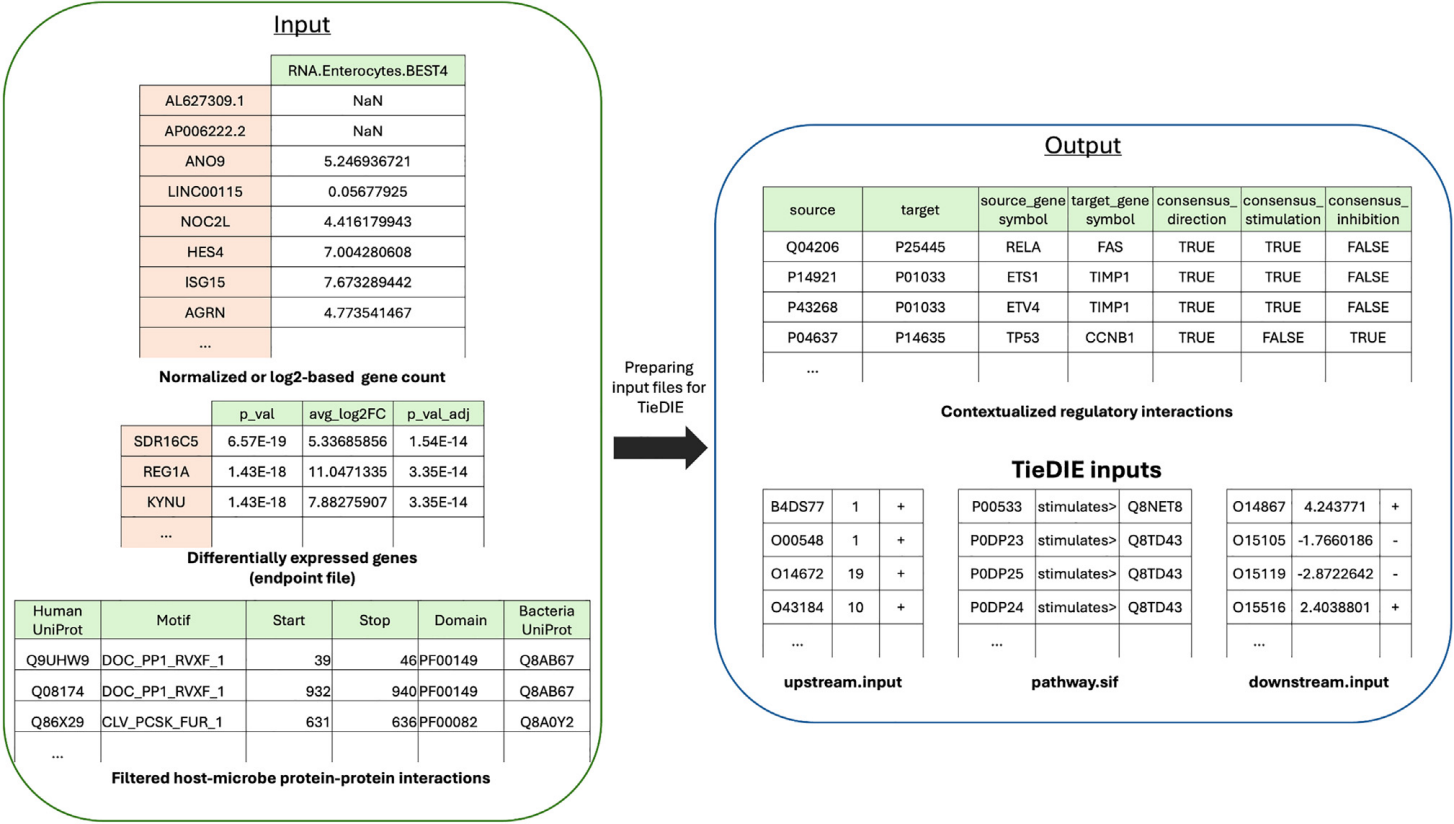
Integrating transcriptomic data with the predicted host-microbe interactions to prepare the input files for the TieDIE analysis
7. Running the TieDIE analysis (tiedie.py).
  ***Note:*** TieDIE (Tied Diffusion Through Interacting Events) is a computational tool for network analysis aimed at discovering causal relationships within biological networks.^13^ It takes the upstream, pathway and downstream inputs, described in Step 6 (Figure 7).
  a. Run the script with the necessary command-line arguments.
    >cd MicrobioLink2/workflow
    >python tiedie.py --network “case_study_output/output/TieDIE/usecase_pathway.sif” --up_heats “case_study_output/output/TieDIE/usecase_upstream.input” --down_heats “case_study_output/output/TieDIE/usecase_downstream.input” --output_folder “case_study_output/output/”
    ***Note:*** The "**--upheats**" parameter is a required argument that specifies the path to a file containing upstream heat values (Step 6). This file contains the gene/protein identifier (UniProt or gene symbol) in the first column; heat values in the second column representing the impact of microbial interactions on upstream host proteins, quantified by the number of bacterial proteins targeting each molecule; and sign indicating whether the interactions’ effect is positive (+) or negative (−).
    ***Note:*** The “**--down_heats**” parameter is a required argument that specifies the path to a file containing downstream heat values (Step 6). This file provides the transcription factors (TF) identifier (UniProt or gene symbol) in the first column; activity data on downstream TFs in the second column, showing how microbial interactions may indirectly influence gene regulation; and sign indicating whether the TF activates (+) or repress (−) target gene expression.
    ***Note:*** The “**--network”** parameter is a required argument that specifies the path to a .sif network file having a specific structure with three columns. The first column (<source>) describes the identifier of the source node (UniProt or gene symbol); The second column (<interaction>) specifies the type of relationship or interaction between the nodes; and the third column (<target>) describes the identifier of the target node (UniProt or gene symbol). This file represents a curated pathway network to be used for pathway search and analysis, formatted with specific directional relationships between nodes.
    ***Note:*** The **--output_folder** parameter is a required argument that specifies the directory path where the program will save all output files.
    ***Note:*** TieDIE results in two main outputs, a network file (“*tiedie.cn.sif*”) that contains information on source and target nodes, relationship and layer; and a Linker Heats file (“*heats.NA*”) that provides information on the linker nodes’ heat values in the subnetwork (Figure 7).
    ***Note:*** The code runs several external scripts in the background (kernel.py; kernel_scipy.py; master_reg.py; permute.py; ppr.py; and tiedie_util.py) that are in the Github repository – ‘*workflow/TieDie/TieDie*’ – these should be placed in the same folder as ‘*tiedie.py’* on the user’s computer.
    **CRITICAL:** After running tiedie.py you must delete the tiedie_kernel.pkl file which is created automatically and will be found in your script folder. It is important to be able to run the script again without error, details are described in the “troubleshooting 4” section.
    ***Note:*** If the output file is empty after running the script, it may occur if the input files do not have unified identifiers, such as UniProt IDs, across all files; details are described in the “troubleshooting 7” section.
    ***Note:*** The script results in several additional outcomes, but further steps do not use them directly. These are the following: Node Statistics (“*node.stats*”); Cytoscape Node Types (“*node_types.NA*”); Individual Source Neighborhood Files; Report (“*report.txt*”); Score Distribution (“*score.txt*”); Permuted Scores (“*permuted_scores.txt*”). Further details are available here: https://sysbiowiki.soe.ucsc.edu/tiedie.

**Figure 7:**
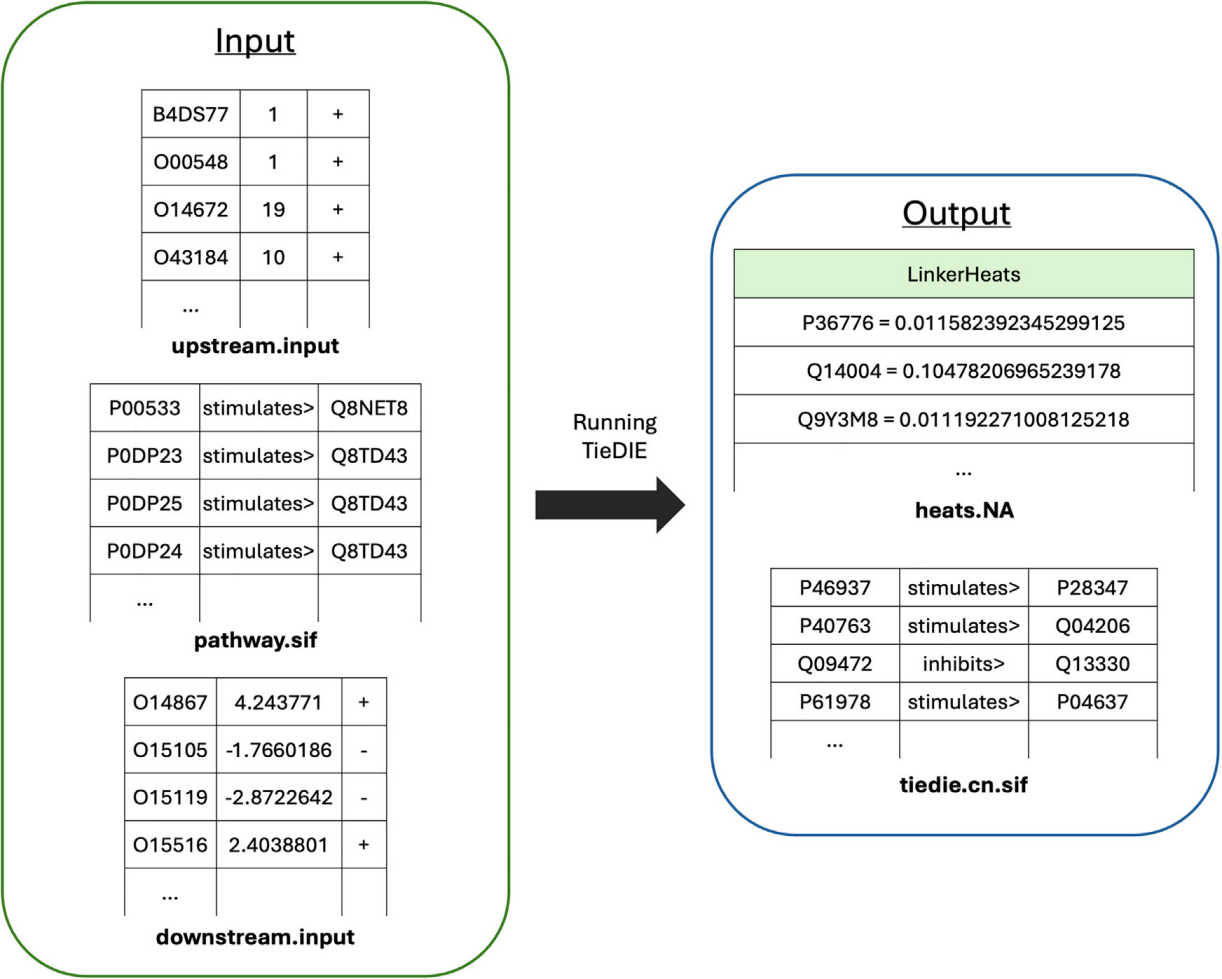
Running TieDIE to identify the signaling network potentially influenced by bacteria and quantify signal propagation through nodes in the network
8. Processing TieDIE output files (tiedie_output_processing.py).
  ***Note:*** After running the TieDIE analysis, the output files require processing to generate interpretable data formats for visualization and downstream analysis. This step combines TieDIE outputs (“*tiedie.cn.sif*” and “*heats.NA*” - Step 7), the regulator-target network file (“*contextualised_regulator-target_network.txt*” - Step 6), the filtered HMI file (Step 5), and the endpoint (Figure 8).
  ***Note:*** By combining the input files, users can visualize and analyze how microbial interactions influence host cellular processes, making it easier to identify key pathways and regulatory targets affected by microbial activity. The main processed outputs include a network file and a node attribute file, which facilitate pathway visualization in Cytoscape or other network analysis tools.
  a. Run the script with the necessary command-line arguments.
    >cd MicrobioLink2/workflow
    >python tiedie_output_processing.py --tiedie_file “case_study_output/output/TieDIE/tiedie.cn.sif” --heats_file “case_study_output/output/TieDIE/heats.NA” --hmi_file “case_study_output/output/HMI/IUPred/BT_enterocyte_idr_cd_usecase.csv” --tf_tg_file “case_study_output/output/TieDIE/contextualised_regulator-deg_network.txt” --endpoint_file “case_study_input/input/human_transcriptomics/Enterocytes BEST4_degs_fc05.csv” --sep_endpoint "," --endpoint_pvalue_column 2 --endpoint_value_column 3 --network_output “case_study_output/output/TieDIE/usecase_final_network.txt” --node_attr_output “case_study_output/output/TieDIE/usecase_node_table.txt”
    ***Note:*** The "**--tiedie_file**" parameter is a required argument that specifies the path to the "*tiedie.cn.sif*" file (Step 7). This file captures the entire signaling network, detailing connections from upstream microbial targets through intermediary host proteins to downstream regulatory targets. The default file name is set to "*tiedie.cn.sif*", but the user can specify a different file path or name as needed.
    ***Note:*** The "**--heats_file**" parameter is a required argument that specifies the path to the "*heats.NA*" file (Step 7). This file contains node heat values that indicate the relative influence or activity level of each node in the network. The default file name is set to "*heats.NA*", but the user can specify a different file path or name as needed.
    ***Note:*** The "**--hmi_file**" parameter is a required argument that specifies the path to an existing file containing host-microbe interaction prediction data (Step 5). This file includes predictions between host and microbial proteins, essential for identifying potential pathways impacted by microbial interactions. In the input file, the first column should be "#Human Protein”, and the sixth column should be "Bacterial Protein". This format is automatically achieved by using the output of idr_prediction.py script, described in Step 5.
    ***Note:*** The "**--tf_tg_file**" parameter is a required argument that specifies the path to the *contextualized_regulatory_network.txt* (Step 6). This file shows how TFs regulate host gene expression, enhancing understanding of microbial impacts on gene expression.
    ***Note:*** The "**--endpoint_file**" parameter is a required argument that specifies the file path to an endpoint file. This file described the target genes of interest, such as genes with differential expression, that are relevant to specific host responses.
    ***Note:*** The "**--endpoint_pvalue_column**" parameter is an optional argument that specifies the column number in the endpoint file containing the adjusted p-value or FDR value for differentially expressed genes. This column is typically used to filter genes based on statistical significance, retaining only those with p-values below a certain threshold (e.g., 0.05). If not provided (e.g. the endpoint file is not a DEG table but a list of genes of interest), the script will include all genes in the endpoint file. User provided *“-- endpoint_pvalue_column 2*” would refer to the second column.
    ***Note:*** The "**--endpoint_value_column**" parameter is a required argument that specifies the column number in the endpoint file containing the fold change or expression values for (differentially) expressed genes. This data is commonly used in downstream analyses to assess the direction and magnitude of gene expression. User provided *“-- endpoint_value_column 3*” would refer to the third column.
    ***Note:*** The "**--sep_endpoint**" parameter is a required argument that specifies the separator (e.g., ";", "\t", or "|") used in the endpoint data file, enabling the program to correctly parse the data.
    ***Note:*** The "**--network_output**" parameter is a required argument that specifies the path and filename for the network output file.
    ***Note:*** The "**--node_attr_output**" parameter is a required argument that specifies the path and filename for saving the node attribute output.
    ***Note:*** The following error message may appear if the mygene package is not installed properly: ‘AttributeError: partially initialized module 'charset_normalizer' has no attribute 'md__mypyc'’. Further details and solutions for this issue are provided in the “troubleshooting 6” section.
    ***Note:*** If the output file is empty after running the script, it may occur if the input files do not have unified identifiers, such as UniProt IDs, across all files; details are described in the “troubleshooting 7” section.

**Figure 8:**
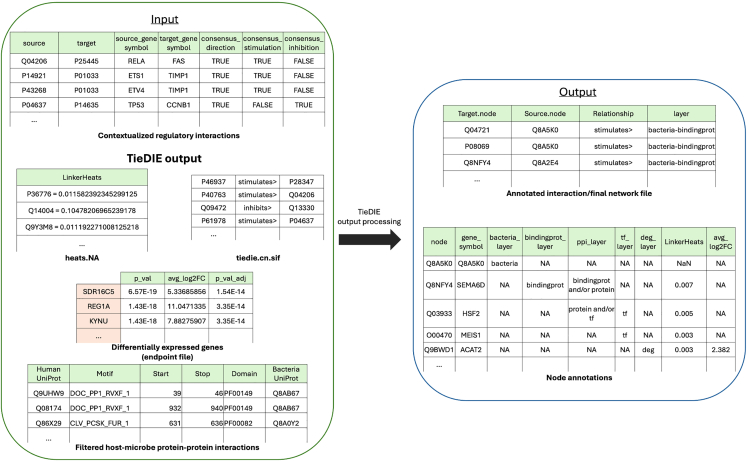
Preparing annotated interaction and node files for network visualization in Cytoscape

### Functional analysis

**Timing: 5–10 min(depending on the size of data)**

The functional analysis step is an addition to the MicrobioLink pipeline, providing insight into the biological interpretation of the predicted host-microbe interaction network. By performing enrichment analysis on proteins potentially affected by microbial interactions, this step allows researchers to identify key biological processes that are likely influenced by microbial activity, potentially highlighting the molecular mechanisms behind host responses and disease processes.

**Figure 9:**
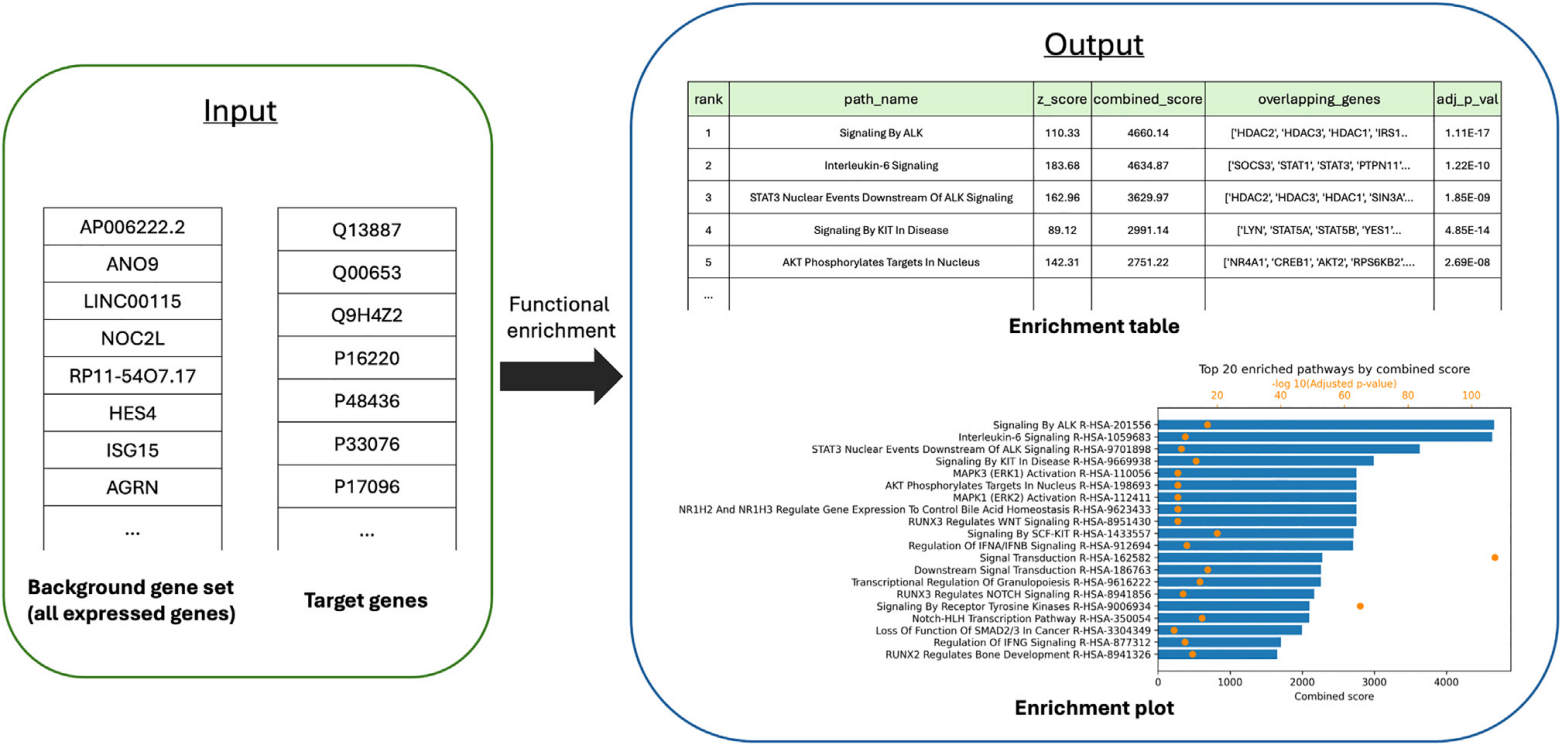
Functional enrichment analysis using the enrichR Python package

9. Enrichment analysis (enrichr_id_database_ranking.py).
  ***Note:*** This step performs functional enrichment analysis using the gget library. It reads background and target gene lists and performs the analysis using the Enrichr database. It generates a bar plot of the top enriched pathways based on either a combined score – natural log of the p-value multiplied by the z-score –, or adjusted p-value and saves the results to an output table and a plot (Figure 9).
  ***Note:*** Functional enrichment is conducted on two levels—either directly on the human proteins targeted by microbial proteins or on the downstream signaling network inferred by TieDIE. This flexibility allows for a broad or focused approach depending on the study goals.
  ***Optional:*** This step may be skipped if the user does not want to perform enrichment analysis on the multi-layered network.
  a. Run the script with the necessary command-line arguments.
    >cd MicrobioLink2/workflow
    >python enrichr_id_database_ranking.py --background_gene_list “case_study_input/input/human_transcriptomics/enterocyte_colon_CD_expressed_genes.csv” --sep “;” --id_background "genesymbol" --target_gene_list “case_study_output/output/TieDIE/tiedie.cn.sif.txt” --analysis_level “TieDIE” --output_image “case_study_output/output/Enrichment_analysis/gget_enrichr_results_reactome_usecase_tiedie.png” --output_file “case_study_output/output/Enrichment_analysis/gget_enrichr_results_reactome_usecase_tiedie.csv” --database “Reactome_2022” --ranking “combined_score”
    ***Note:*** The "**--background_gene_list**" parameter is a required argument that specifies the path to a file containing the background gene list (UniProt or gene symbol IDs) in the first column. This list contains all genes expressed in the dataset, providing a reference set for enrichment analysis. The background set helps define the scope of comparison for identifying significantly enriched pathways or gene sets.
    ***Note:*** The "**--sep**" parameter is a required argument that specifies the separator (e.g., ";", "\t", or "|") used in the background gene file. This allows the program to correctly parse the data, as different files may use different delimiters.
    ***Note:*** The "**--id_background**" parameter is a required argument that specifies the type of identifier (UniProt or genesymbol) used for the background genes in the background gene file.
    ***Note:*** The "**--target_gene_list**" parameter is a required argument that specifies the list of target genes (UniProt or gene symbol IDs). This list includes genes specifically affected by microbial interactions, either as direct microbial targets or as downstream elements in the TieDIE-inferred network.
    ***Note:*** The "**--analysis_level**" parameter is a required argument that specifies the stage at which the enrichment analysis will be performed. The argument expects a choice between two options. The "*HMI*" option performs enrichment directly on human proteins that are targeted by bacterial interactions (Step 5), useful for understanding host-microbe interactions at a high level. The "*TieDIE*" option focuses on the downstream signaling network inferred through the TieDIE pathway analysis (Step7), highlighting signaling pathways impacted by initial bacterial interactions.
    ***Note:*** The "**--output_image**" parameter is a required argument that specifies the path and filename for saving the output image generated by the enrichment analysis. This image typically visualizes the top enriched pathways, providing an accessible, graphical summary of the most significant pathways affected by the target genes. The file format should generally be suitable for graphical data, such as .png, .jpg, or .pdf, depending on user preference.
    ***Note:*** The "**--output_file**" parameter is a required argument that specifies the path and filename for saving the results of the enrichment analysis in a text format.
    ***Note:*** The "**--database**" parameter is a required argument that specifies the reference database for the enrichment analysis. This parameter enables users to choose a relevant database from which pathways or gene sets are selected to perform the analysis. The parameter supports specific shortcuts for commonly used databases, each tailored to different types of biological questions, such as “*GO_Biological_Process_2021*” for annotations or “*ChEA_2016*” for transcription. Visit https://maayanlab.cloud/Enrichr/#libraries for further databases. The default reference database is “Reactome_2022”, the most up-to-date version of the database (September 2024).
    ***Note:*** The **--ranking** parameter is a required argument that specifies the metric used to rank pathways in the enrichment analysis plot. If not provided, the script will use "combined_score" as the default ranking metric, which is commonly used method to combine the significance (p-value) and strength (z-score) of enrichment. The argument expects a string, with two options available. The default "*combined_score*" option ranks pathways by the combined score, calculated as the natural log of the p-value multiplied by the z-score. This score highlights pathways that are both statistically significant and highly enriched. The "*adj_p*" option, however, ranks pathways by the adjusted p-value, which is a more conservative approach, focusing strictly on statistical significance after correction for multiple testing.
    ***Note:*** The following error message may appear if the mygene package is not installed properly: ‘AttributeError: partially initialized module 'charset_normalizer' has no attribute 'md__mypyc'’. Further details and solutions for this issue are provided in the “troubleshooting 6” section.
    ***Note:*** If the output file is empty after running the script, it may occur if the input files do not have unified identifiers, such as UniProt IDs, across all files; details are described in the “troubleshooting 7” section.

## Expected outcomes

We performed a case study focusing on the effects of the probiotic *Bacteroides thetaiotaomicron* (Bt)-derived extracellular vesicles (BEVs) on host cellular signaling in inflammatory bowel disease (IBD). Extracellular vesicles are small membrane-coated structures, secreted by bacteria, that play an important role in intercellular communication, including host-microbe interactions. Previous research has shown that Bt can ameliorate inflammation in mouse models of IBD, making it a promising candidate for studying its underlying molecular mechanisms that may help to identify promising therapeutic targets for IBD.

To explore these effects, we used proteomic data from Bt BEVs^1^ and human gene expression data from colonic enterocytes of Crohn’s disease (CD) patients obtained from public single cell transcriptomic data (https://singlecell.broadinstitute.org; ID: SCP259). The case study input and output files are available in the GitHub repo – “*case_study_input”* and “*case_study_output”* folders. The pipeline generated three main outcomes, each offering insights into different aspects of the host-microbe interaction.

### Host-microbe protein-protein interaction network

**Figure 10:**
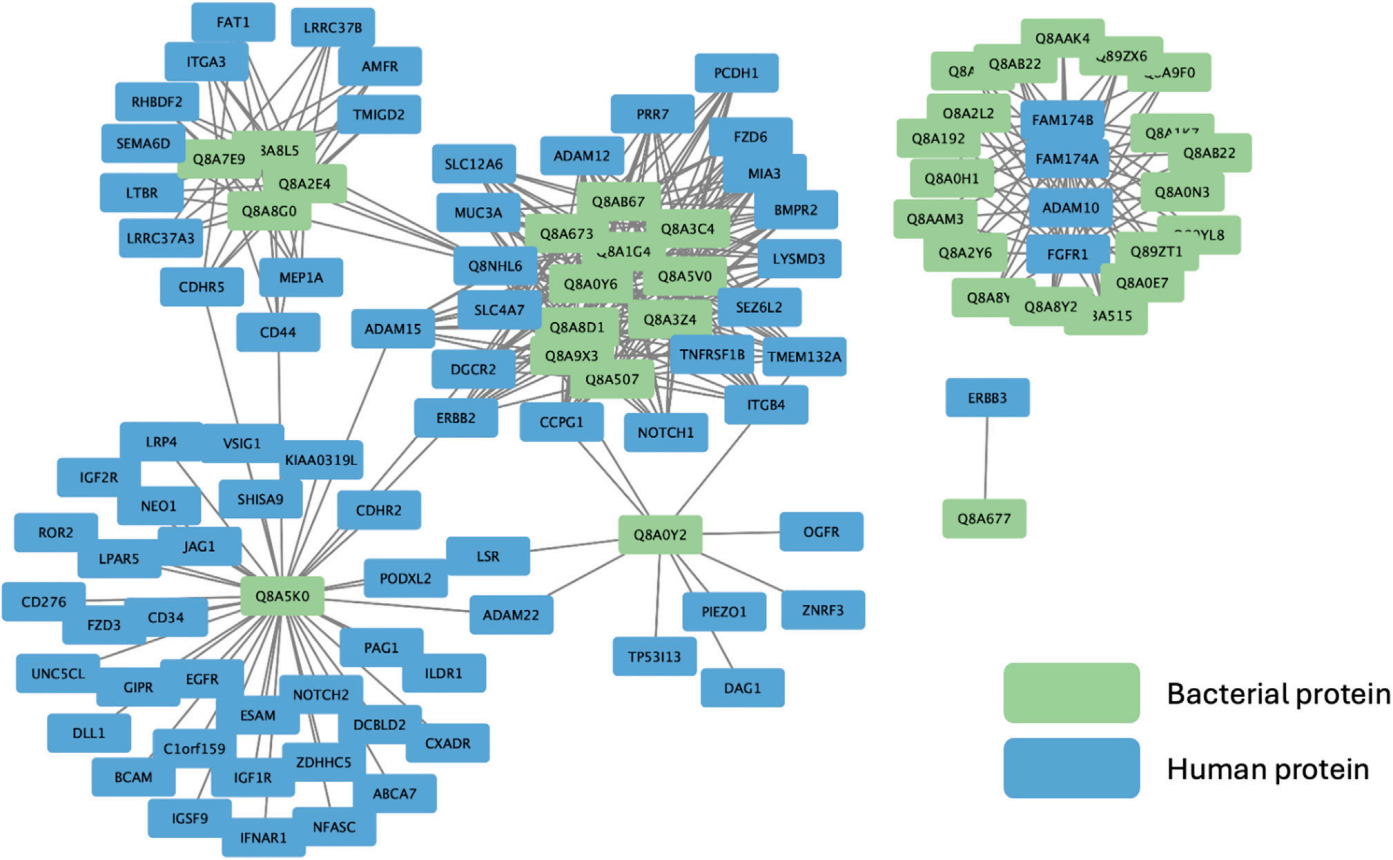
Predicted protein-protein interactions between *B. thetaiotaomicron*-derived extracellular vesicles and colon enterocyte cells in Crohn’s disease

The first outcome is a predicted network of protein-protein interactions between bacterial proteins from Bt and human proteins, connected based on DMIs (Figure 10). The interaction network, detailed in Step 2, reveals specific contact points where Bt proteins may influence host proteins, suggesting potential molecular sites where microbial effects on host cellular processes may begin.

### Multi-layered protein-protein interaction network

**Figure 11:**
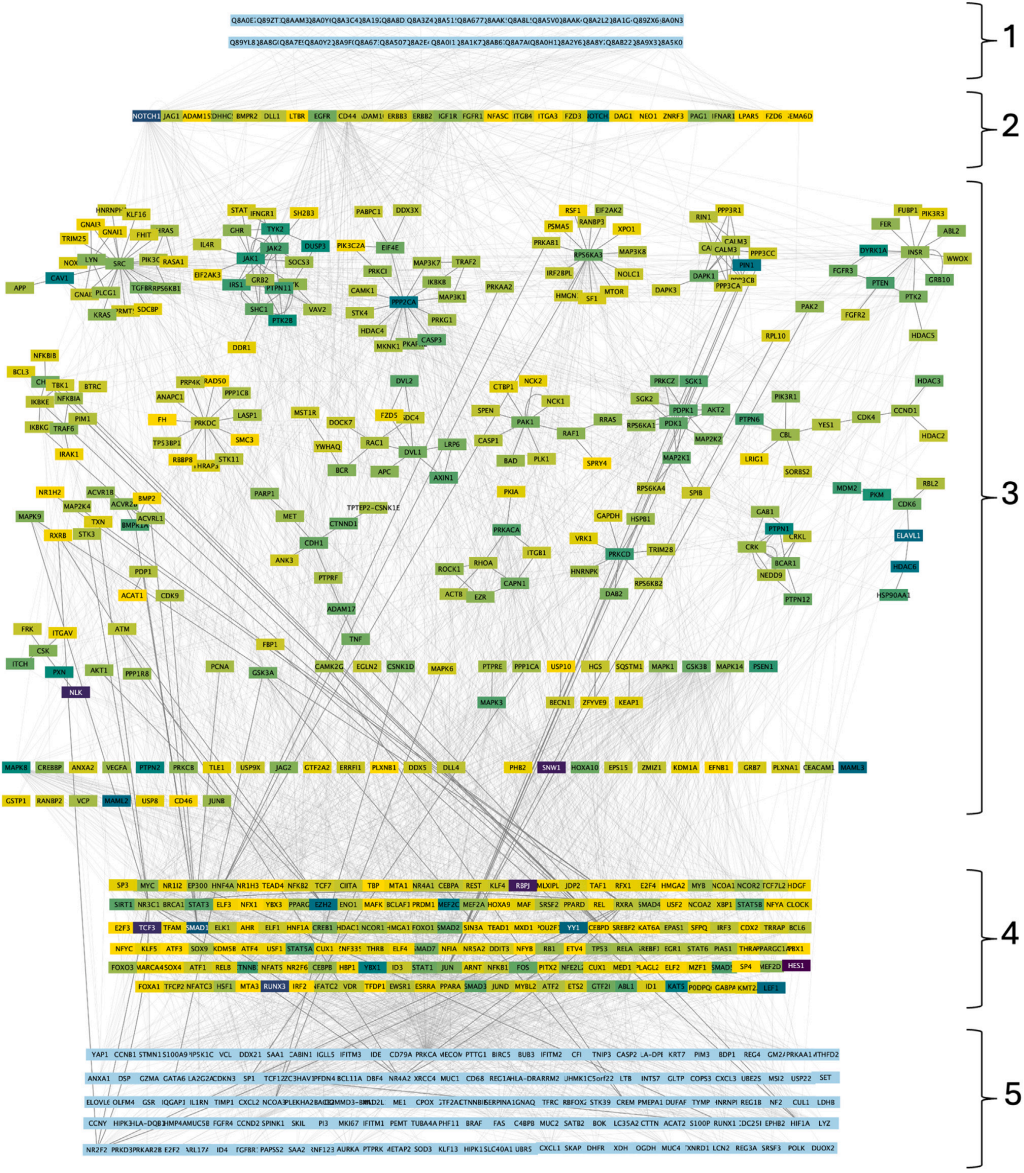
The potential downstream effect of proteins of *B. thetaiotaomicron*-derived extracellular vesicles on Crohn’s disease patient-derived colon enterocytes Nodes are coloured to represent heat levels based on a yellow (low heat values)-to-purple (high heat values) scale. Blue nodes depict proteins with no heat values, including bacterial proteins and differentially expressed genes. Intermediate signaling proteins are clustered using the clusterProfiler Cytoscape package. Numbers show the different set of proteins and genes in the multi-layered network: 1. Bacterial proteins 2. Human membrane proteins 3. Intermediate protein-protein interaction signaling network 4. Transcription factors 5. Differentially expressed genes.

The second outcome is a multi-layered network that extends the host-microbe interactions by linking Bt-derived proteins to differential gene expression patterns. This network, detailed in Step 3 and visualized in Cytoscape using the clusterProfiler package, is shown in Figure 11. This outcome reveals how Bt BEVs could impact gene regulation differently in healthy vs. inflamed conditions. This allows researchers to identify pathways and cellular functions that are probably influenced by microbes, helping to discover potential therapeutic targets.

### Enrichment analysis of affected pathways

**Figure 12:**
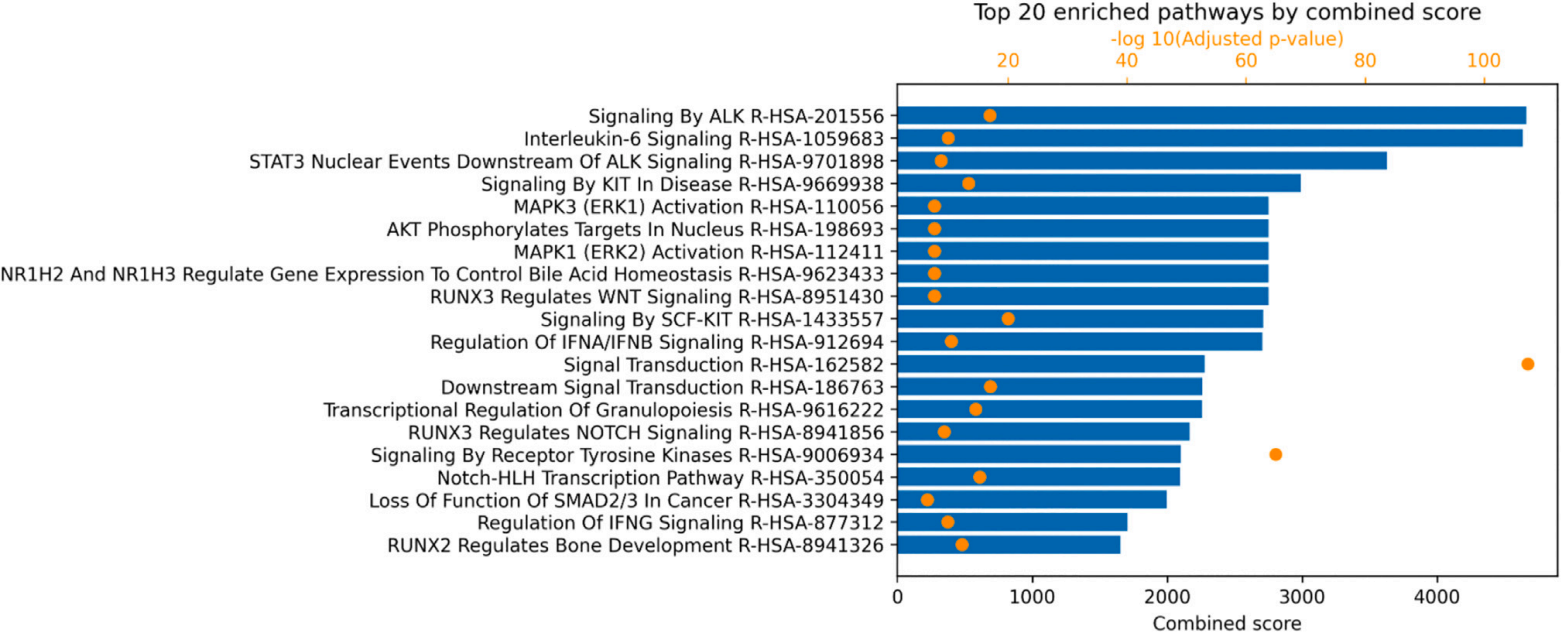
The top 20 enriched pathways based on combined scores for *B. thetaiotaomicron* and colon enterocyte cells in Crohn’s disease based on the Reactome database

The third outcome is a bar plot of the top 20 enriched pathways influenced by Bt-BEV interactions, based on combined scores, detailed in Step 9. This plot, shown in Figure 12, is automatically generated by the pipeline running the enrichment analysis. The output offers insights into mechanisms through which Bt BEVs could potentially mitigate inflammation or promote immune balance in IBD.

## Limitations

While MicrobioLink offers a robust framework to integrate human transcriptomics and bacterial proteomics data for predicting host-microbe interactions, several limitations should be considered when interpreting the results. These limitations show the areas where future improvements could enhance the accuracy and applicability of the pipeline.

### Dependence on pre-processed input data

MicrobioLink requires pre-processed multi-omic data, such as normalized gene count matrices for human data and UniProt IDs for bacterial proteomics, to ensure compatibility. When working with similar datasets, researchers may need to format their multi-omic data according to the specific requirements outlined in the case study.

### Reliance on domain-motif interactions from the ELM database

The pipeline uses domain information from the Pfam database and DMIs from the ELM database, which lack information about bacteria-specific domain interactions. This limitation could lead to missed predictions of interaction sites. To address this, the integration of the AlphaFold tool, a deep learning model for predicting protein structures, is currently under development potentially improving prediction accuracy in future versions.

### Dependence on public databases for functional analysis

While databases, like Reactome or KEGG, provide the advantage of quickly associating genes with functional terms, they also carry the risk if certain pathways are overrepresented or underrepresented. To mitigate this bias, users are encouraged to integrate additional annotation sources, reducing dependence on a single database and enhancing the robustness of the results.

### Limited protein-protein interactions

The current version of MicrobioLink focuses on protein-protein interactions. However, many host-microbe interactions are mediated by metabolites, which play crucial roles in communication and signaling between the host and microbial communities.^14^ The integration of metabolite data analysis into the pipeline is currently under development.

These limitations provide context for interpreting the results generated by MicrobioLink. While the pipeline is a versatile tool for studying host-microbe interactions, addressing these limitations through future developments could enhance its accuracy and broader applicability.

## Troubleshooting

### Problem 1

Some software or packages are unavailable, leading to errors.

### Potential solution

This error may arise at any step (Step 1-9), as each script relies on external package dependencies. To resolve this issue, ensure all steps are executed within the designated computing environment, which should be correctly set up according to the instructions in the “before you begin” section. Verifying that the required packages are installed and accessible in this environment can prevent these availability issues.

### Problem 2

One or multiple paths to the directories, source files, or the output folder the user defined do not exist – *“python: can’t open file 'path/to/file’': [Errno 2] No such file or directory”*

### Potential solution

This error may occur at any step (Step 1-9). Verify that you have provided the paths to the folders where your source files are located, respectively, without including the file names at the end of the path. Ensure that the output directory you defined exists. For more detailed information, refer to the GitHub documentation (key resources table) or the functions’ documentation in Python.

### Problem 3

Missing parameter definition:

 Error: usage: script.py [-h] -p1 PARAMETER1 -p2 PARAMETER2 -p3 PARAMETER3

 script.py: error: the following arguments are required: -p3/--parameter3.

### Potential solution

This error may arise at any step (Step 1-9) when running the scripts from the Terminal/Command Line if a required parameter is not defined. The help message, which can be accessed by running *script.py -h*, provides additional guidance on the parameters required for each step.

### Problem 4

Specific error message while running TieDIE in Step 7 – ‘*Error: the universe of gene/node labels in the network file doesn’t match the supplied kernel file*!’

### Potential solution

This error typically occurs when there is a mismatch between the gene or node labels in the network file and the kernel file used in the TieDIE analysis, often due to a prior unsuccessful run or an outdated intermediate file. To resolve this issue, delete the .pkl file in the ‘*workflow/TieDie/TieDie*’ folder. This file is a cached intermediate output that stores network data, and deleting it allows TieDIE to generate a new version aligned with the current network and kernel files.

### Problem 5

Error message: cannot import name 'Mapping' from 'collections'.

### Potential solution

This error is typically caused by an incompatibility issue with the *collections* Python package and may occur in Step 4 during the *in silico* host-microbe interaction prediction. To resolve this issue, use Python 3.9, as the *collections* package used by the pyfasta library is compatible with this version.

### Problem 6

AttributeError: partially initialized module 'charset_normalizer' has no attribute 'md__mypyc'

### Potential solution

This error is typically caused by an incorrect installation of the *mygene* Python package, and it can occur in steps that depend on this package (Step 2, Step 8, and Step 9). Reinstalling the *charset-normalizer* package, a dependency of *mygene*, usually resolves the issue. To fix this, open the Terminal/Command Line and run the following command:

>pip install --force-reinstall charset-normalizer==3.1.0

### Problem 7

The output file is empty.

### Potential solution

This issue often arises from incorrect input formatting, mismatched molecular IDs or a lack of predicted interactions. Follow these steps to troubleshoot:

**Incorrect input formatting:** This error may arise at any step (Step 1-9), verify that the input files follow the expected structure and format as described in each step, with gene identifiers in the correct columns, proper separators, and accurate file paths.

**ID issues:** This issue may occur in Step 6, Step 7, Step 8 or Step 9; when merging multiple input files, it is essential that each file uses the same type of identifiers (e.g., UniProt IDs) for successful integration. Although the scripts are designed to be flexible, they require consistency in the identifier format across all input files to ensure proper alignment and merging of data. Before running the pipeline, verify that each input file uses a uniform ID format. If necessary, convert IDs in one or more input files to match the format used by the others. You can use tools like UniProt’s ID mapping service to standardize identifiers.

**No significant interactions detected:** If the input data is formatted correctly, an empty output may indicate that no significant interactions met the criteria for inclusion in the output in Step 4 or Step 5. To confirm, consider setting lower thresholds or checking intermediate files to ensure that earlier steps generated expected results.

## Resource availability

### Lead contact

Further information and requests for resources should be directed to and will be fulfilled by the lead contact, Tamas Korcsmaros (t.korcsmaros@imperial.ac.uk).

### Technical contact

Questions about the technical specifics of performing the protocol should be directed to and will be answered by the technical contact, Lejla Gul (l.potari-gul@imperial.ac.uk).

### Materials availability

The case study used human single-cell transcriptomics published by Kong et al., 2023,^3^ while the bacterial proteins derived from proteomic data published in Gul et al.^1^

### Data and code availability

The code generated during this study is available on GitHub (https://github.com/korcsmarosgroup/MicrobioLink2/tree/main/workflow) and Zenodo (https://doi.org/10.5281/zenodo.14336678). The MicrobioLink tool source code was previously published^15^ and is available alongside extensive documentation on GitHub.

## Acknowledgments

The work of L.G., B.B., and T.K. was supported by the UKRI BBSRC Institute Strategic Programme Food Microbiome and Health BB/X011054/1 and its constituent project BBS/E/F/000PR13631. T.K. was also supported by the NIHR Imperial Biomedical Research Centre Organoid Facility. The views expressed are those of the authors and not necessarily those of the NIHR or the UK Department of Health and Social Care. A.J.E. is supported by the Semmelweis Science and Innovation Fund, Semmelweis Young Researchers Research Grant (STIA-MEC 2022); the Federation of European Microbiological Societies, FEMS Research and Training Grant (ID 4695); and the Kerpel-Fronius Ödön Talent Program, Kerpel Scholarship. D.M. acknowledges financial support from Imperial College London through an Imperial College Research Fellowship grant award. M.M. was supported by the BBSRC Norwich Research Park Biosciences Doctoral Training Partnership (BB/M011216/1 and BB/S50743X/1). D.M. and T.K. were supported by the BBSRC Gut Microbes and Health Institute Strategic Programme BB/R012490/1 and its constituent projects BBS/E/F/000PR10353 and BBS/E/ F/000PR10355. The graphical abstract was created using Biorender.com.

## Author contributions

Script implementation, L.G., A.J.E., T.T., D.M., M.O., B.B., and M.M.; pipeline testing, L.G., A.J.E., T.T., M.O., and E.W.; case study implementation, A.J.E.; manuscript writing, L.G. and A.J.E.; reviewing, T.K.; supervision, T.K. All authors approved the final version.

## Declaration of interests

The authors declare no competing interests.
